# Taurine's health influence on Japanese high school girls

**DOI:** 10.1186/1423-0127-17-S1-S47

**Published:** 2010-08-24

**Authors:** Megumi Ishikawa, Shiho Arai, Mio Takano, Atsumi Hamada, Kazuhiro Kunimasa, Mari Mori

**Affiliations:** 1 Super Science Course, Mukogawa Women's University Senior High School, Nishinomiya, 6638143, Japan; 2 Institute for World Health Development, Mukogawa Women's University, Nishinomiya, 6638143, Japan

## Abstract

**Background:**

The prevalence of metabolic syndrome (MS) in children and adolescents has been increasing at an alarming rate. MS risks during childhood and adolescence adversely affect health conditions in later life. Thus, the characterization of their MS risks is a critical research field. The aims of this study are to survey the health status of Japanese adolescent females, a poorly characterized population, and to investigate the potential relationship between their MS risks and dietary factors like potassium (K) and taurine.

**Methods:**

Anthropometric characteristics of 243 healthy school girls aged 13 to 18 years were measured. Serum levels of triglycerides, total cholesterol and high-density lipoprotein (HDL), and plasma levels of glucose and insulin were analyzed in fasting blood samples. We assessed overweight, disturbed lipid prolife, higher blood pressure (hBP) and higher plasma glucose (hGlc) levels as indicators of MS risks. The relationships between MS risks and urinary K or taurine excretion were investigated by dividing into higher and lower groups at medians of their urinary excretions.

**Results:**

Half of junior high school (JHS) and one-quarter of senior high school (SHS) girls had at least one MS risk. The quite common risk was hGlc, the rates being 21% in JHS girls and 14% in SHS. The prevalence of being overweight and obesity were only small portions, the rate being 0% and 0% in JHS girls, and 10% and 1% in SHS, respectively. Substantial differences in the prevalence of hBP were observed between JHS (22%) and SHS (4%) girls. Furthermore, higher urinary K excretion group showed a significant decrease in triglyceride level (*P *= 0.03) and increase in HDL level (*P *= 0.003) compared with the lower. Also, the higher urinary taurine excretion group exhibited a significant reduction in triglyceride level (*P *= 0.04) compared with the lower.

**Conclusions:**

These results indicate that control of plasma glucose level rather than body weight is a crucial task in Japanese pubertal girls, and that a dietary habit rich in K and taurine could improve their lipid profile. Nutritional education based on these findings would help to prevent the future development of MS in Japanese female adolescents.

## Background

Metabolic syndrome (MS) is a cluster of risk factors for type 2 diabetes mellitus (T2DM) and cardiovascular diseases (CVDs), which includes obesity, insulin resistance, hypertension and dyslipidemia. A recent large cohort study on school children revealed the significant association of higher MS risks during childhood with increased risks for CVDs in adulthood [1]. This implies not only that the origins of T2DM and CVDs begin in childhood and adolescence, but also that MS risks in childhood and adolescence adversely affect health conditions in later life. Given that the prevalence of MS in children and adolescents has been increasing at an alarming rate [2,3], the worldwide epidemic of MS could further accelerate in the near future. To prevent such a public health crisis, it is critically important to understand MS risks in children and adolescents as well as adults. However, little is known about health status and MS risks of Asian young population, although those in European and American children and adolescents have been reported [1,2,4].

Our world-wide epidemiological study collaborating with WHO was designated Cardiovascular Diseases and Alimentary Comparison (CARDIAC) study, and has been carried out over the past 25 years in 61 populations in 25 countries to determine the relationship between their MS risks and dietary habits [5-8]. In each population, about 100 males and 100 females aged 48 to 56 years were randomly selected, with the number of participants being over 14,000 in all. The CARDIAC study yielded interesting findings. Namely, the populations with higher sodium (Na) excretion in 24-h urine, correlated with high salt intake, had increased risk for stroke compared with those with lower, whereas the populations with higher taurine excretion, correlated with high seafood intake, had decreased risk for coronary heart disease compared with those with lower [7,9,10]. Also, increased excretion of urinary potassium (K), an indicator for vegetables and fruits intakes, was reported to inversely correlate with body mass index (BMI) and blood pressure (BP) [11]. These findings indicate that dietary habit beneficially or adversely affect health status and MS risks. Thus, the characterization of dietary habits may provide a clue to properly control MS risks. Considering that the recording of accurate dietary history is difficult for participants, 24-h urine analysis would be a practical and reliable tool for the characterization of dietary habits. In this study, we conducted a health survey of 243 healthy school girls aged 13-18 years at Mukogawa High School in Nishinomiya, Japan, to obtain baseline data on the health status of Japanese adolescent females, and then investigated the potential relationship between their MS risks and dietary habits by 24-h urine analysis in combination with anthropometric measurements and blood chemical analysis.

## Methods

### Subjects

A total of 243 healthy school girls aged 13 to 18 years participated in this health survey held at Mukogawa junior and senior high school in Nishinomiya, Japan, from 2007 to 2008. The participants were composed of 67 junior high school (JHS) girls and 176 senior high school (SHS) ones. The study design was approved by the research ethics committee of the Mukogawa Women's University.

### Health examination

Anthropometric characteristics, including height, weight and abdominal circumference (AC), were measured. BMI was calculated as (body weight [kg])/(height [m])^2^. BP was measured twice with an automated sphygmomanometer (HEM-970; OMRON, Kyoto, Japan) in sitting position after over 10 minutes rest and the average was used in this study. According to the criteria of MS risks in JHS and SHS students established by the Ministry of Health, Labour and Welfare in Japan, higher BP (hBP), overweight, obesity and increased AC were defined as follows: hBP, systolic BP (SBP) ≧ 125 mmHg and/or diastolic BP (DBP) ≧ 70 mmHg for JHS students and SBP ≧ 130 mmHg and/or DBP ≧ 80 mmHg for SHS ones; overweight, BMI ≧ 25; obesity, BMI ≧ 30; increased AC, AC (cm)/height (cm) ≧ 0.5 or AC ≧ 80 cm for JHS students and AC ≧ 90 cm for SHS ones. Also, the rate of thinness (BMI < 18.5) in the participants was determined according to the criterion set by the Japan Society for the Study of Obesity.

### Blood chemical analysis

Blood samples were obtained after a more than 3-h fasting. Serum levels of triglycerides, total cholesterol and high-density lipoprotein (HDL), and plasma levels of insulin and glucose were determined by SRL, Inc (Tokyo, Japan). According to the criteria of MS risks in JHS and SHS students established by the Ministry of Health, Labour and Welfare in Japan, higher plasma glucose (hGlc) and disturbed lipid profile (dLP) were defined as follows: hGlc, fasting plasma glucose ≧ 110 g/ml; dLP, serum triglycerides ≧ 120 mg/dl and/or serum HDL < 40 mg/dl for JHS students and serum triglycerides ≧ 150 mg/dl and/or serum HDL < 40 mg/dl for SHS ones. Insulin resistance (homeostasis model assessment of insulin resistance: HOMA-IR) was calculated using the following formula: HOMA-IR = fasting plasma insulin (μIU/ml) × fasting plasma glucose (mg/dl)/405.

### Twenty-four-hour urine analysis

A 24-h urine specimen was collected using a standard aliquot cup that allowed the participants to repeatedly collect an exact portion of voided urine [12]. Taurine concentration (μmol/ml) in the 24-h urine sample was measured by high performance liquid chromatography (HPLC) (GL Sciences, Tokyo, Japan). The concentrations of K (mEq/l) and Na (mEq/l) were determined by ion selective electrodes at SRL, Inc (Tokyo, Japan). Creatinine concentration (mg/l) in the 24-h urine sample was measured by an enzyme method at SRL, Inc. Urinary excretions per day of taurine (μmol/day), K (g/day), Na (g/day) and creatinine (mg/day) were estimated from their urinary concentrations and daily urine volume. Daily intake of NaCl (g/day) was determined from urinary Na excretion. Subjects who had failed to appropriately collect 24-h urine samples were determined by calculating creatinine coefficient (creatinine/body weight) and excluded from this urinary analysis. Two hundred twelve students successfully collected 24-h urine samples. The participants were divided into two (higher or lower) groups at a median of each urinary taurine or K excretion. The differences in the MS risks of their groups were analyzed and compared.

### Statistical analysis

Differences between the two groups were evaluated by student *t*-test. *P *values less than 0.05 were considered as significant for every statistical analysis.

## Results

### MS risks in Japanese JHS and SHS girls

**Figure 1:**
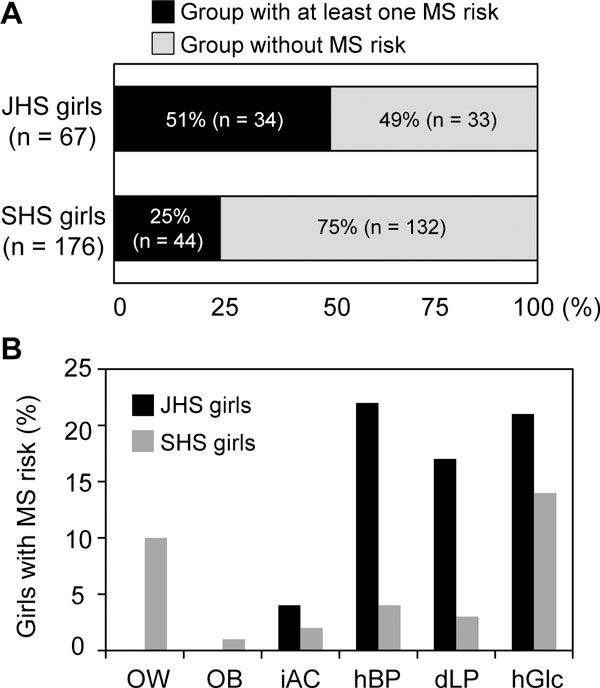
**Rates of JHS and SHS girls with MR risks**. (A) Rates of JSH and SHS girls with or without MS risk in BMI, AC, BP, serum triglyceride and HDL levels, and plasma glucose level. (B) Rates of JSH and SHS girls with each MR risk. OW: overweight, OB: obesity, iAC: increased AC, hBP: higher blood pressure, dLP: disturbed lipid profile, hGlc: higher plasma glucose.

Anthropometric characterization and blood chemical analysis in JHS (n = 67) and SHS (n = 176) girls are shown in Table 1. Since criteria for MS risks, such as increased AC, hBP, dLP and hGlc, are differently defined between JHS and SHS students in Japan, we separately assessed their MS risks. The rate of JHS girls with at least one MS risk reached 51% (n = 34), whereas that of SHS girls was 25% (n = 44) (Figure 1A). The prevalence of hBP, dLP and hGlc were observed in 22%, 17% and 21% of JHS girls, and in 4%, 3% and 14% of SHS, respectively (Figure 1B). The rates of overweight, obesity and increased AC were 0%, 0% and 4% in JHS girls, and 10%, 1% and 2% in SHS (Figure 1B), respectively, whereas those of thinness were 34% in JSH girls and 14% in SHS.

**Table 1:** Anthropometric and blood chemical profiles of junior and senior high school girls

	Junior high school girls (n = 67)	Senior high school girls (n = 67)
Age	14.3 ± 0.7	17.1 ± 0.7*
BMI (kg/m^2^)	19.4 ± 2.1	21.1 ± 2.7*
SBP (mmHg)	107.6 ± 9.8	107.9 ± 10.5
DBP (mmHg)	64.2 ± 7.3	63.8 ± 7.6
Triglycerides (mg/dl)	78.3 ± 47.0	70.2 ± 37.2
T-cho (mg/dl)	171.4 ± 22.5	181.0 ± 31.4
HDL (mg/dl)	60.3 ± 10.6	63.5 ± 12.7
Glucose (mg/dl)	92.9 ± 8.6	92.2 ± 8.0
Insulin (μIU/ml)	14.9 ± 12.2	12.5 ± 11.0
HOMA-IR	3.5 ± 3.2	2.9 ± 2.8

T-cho: total cholesterol.

Values are expressed as means ± SD. *P < 0.05 compared with the value of junior high school girls.

### Inverse correlations between urinary K excretion and dyslipidemic risk in Japanese adolescent girls

**Figure 2:**
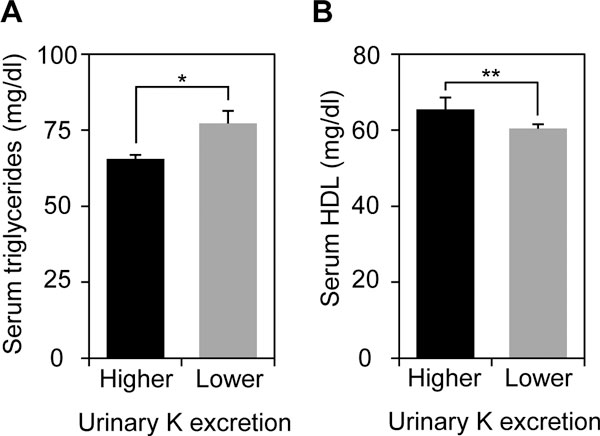
**Inverse correlations between urinary K excretion and dyslipidemic risk in Japanese adolescent girls**. The participants who had successfully collected 24-h urine were divided into higher (n = 105) and lower (n = 107) groups at a median of urinary K excretion. (A) Serum triglyceride level and (B) Serum HDL level were measured in higher and lower urinary K excretion groups. Values are expressed as means ± SE. *P < 0.05, **P < 0.005.

We next measured minerals such as Na and K in their 24-h urine samples to understand dietary habits in Japanese adolescent girls on the basis of evidence showing the correlations between NaCl intake and urinary Na excretion, and between fruits and vegetables intakes and urinary K excretion, respectively. Daily intake of NaCl and urinary K excretion (means ± SD) in JHS girls were 8.3 ± 3.0 g/day and 1.4 ± 0.5 g/day, and those in SHS girls were 7.9 ± 3.2 g/day and 1.4 ± 0.5 g/day, respectively (Table 2). The ratios of Na/K were 4.5 ± 2.6 in JHS girls and 4.0 ± 1.7 in SHS ones. We further divided the students into two (higher or lower) groups at a median (= 1.33 g/day) of urinary K excretion to investigate whether urinary K excretion could correlate with changes in MS risks. It was confirmed that higher urinary K excretion group (n = 105) showed a significant reduction in serum triglyceride level and elevation in serum HDL level compared with the lower group (n = 107) (*P *= 0.03 and 0.003, respectively) (Figure 2A and 2B). No significant differences were observed in other parameters (BMI, SBP, DBP, serum total cholesterol level, plasma glucose and insulin levels, and HOMA-IR) between higher and lower K excretion groups (data not shown).

**Table 2:** Daily intake of NaCl and Urinary profiles in junior and senior high school girls

	Junior high school girls (n = 54)	Senior high school girls (n = 158)
NaCl (g/day)	8.3 ± 3.0	7.9 ± 3.2
K (g/day)	1.4 ± 0.5	1.4 ± 0.5
Na/K ratio	4.5 ± 2.6	4.0 ± 1.7
Taurine (μmol/day)	957.1 ± 717.9	920.7 ± 651.1

Values are expressed as means ± SD.

### Inverse correlations between urinary taurine excretion and dyslipidemic risk in Japanese adolescent girls

**Figure 3:**
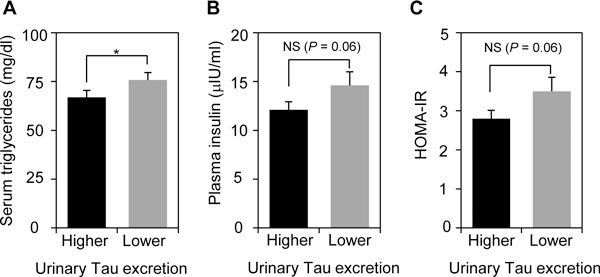
**Inverse correlations between urinary taurine excretion and dyslipidemic risk in Japanese adolescent girls**. The participants who had successfully collected 24-h urine were divided into higher (n = 106) and lower groups (n = 106) at a median of urinary taurine excretion. (A) Serum triglyceride level, (B) plasma insulin level and (C) HOMA-IR were estimated in higher and lower urinary taurine excretion groups. Values are expressed as means ± SE. Tau: taurine, *P < 0.05.

We also estimated taurine excreted in 24-h urine by HPLC analysis and found that urinary taurine excretions (means ± SD) were 957.1 ± 717.9 μmol/day in JHS girls and 920.7 ± 651.1 μmol/day in SHS girls (Table 2). We further divided into two (higher or lower) groups at a median (= 824.5 μmol/day) of urinary taurine excretion to examine whether there were correlations between urinary taurine excretion and changes in MS risks. It was ascertained that higher taurine excretion group (n = 106) showed a significant reduction in serum triglyceride level compared with the lower group (n = 106) (*P *= 0.04) (Figure 3A). Moreover, insulin and HOMA-IR levels had a decreased tendency in higher taurine excreters, although the differences did not reach statistical significance (*P *= 0.06, respectively) (Figure 3B and 3C). There are no significant differences in other parameters (BMI, SBP, DBP, serum total cholesterol and HDL levels, and plasma glucose level) between higher and lower taurine excretion groups (data not shown).

## Discussion

Recent studies demonstrate that the prevalence of MS in children and adolescents living in Europe and North America has dramatically increased in parallel with that in adults [2,3]. There is only limited information available on the prevalence of MS in the Asian young generation, including Japanese, who differs from European and American ones in the component and pathologic mechanisms of MS. These prompted us to survey health status of Japanese school girls and to investigate how their dietary habits affect MS risks.

Anthropometric and blood chemical analyses showed that half of JHS girls and one-quarter of SHS girls had at least one MS risk. The quite common MS risk among JHS and SHS girls was hGlc. These features may reflect excess consumption of sugar-sweetened soft drinks and snacks, decreased physical activity or both. With respect to BMI, the rate of overweight (10%) in SHS girls was found to be nearly equal to the rates of Hong Kong's (9.8%) and British (11.7%) girls, and much lower than those of American (24.0%) and Brazilian ones (15.2%) [13,14], although it seems hard to directly compare their overweight rates because of some differences in age and study period. Given the rate of obesity in JHS and SHS groups were as small as 0% and 1%, respectively, overweight and obesity are likely to be minor risk factors in Japanese female adolescents compared with American and Brazilian ones with a high prevalence of obesity. Also, the substantial difference in the rates of hBP between JHS (22%) and SHS (4%) girls might be attributed to the application of different criterion for hBP established by the Japanese government. Alternatively, such a difference could be accounted for by a higher intake of salty foods in JHS group, because their daily intake of NaCl and Na/K ratio were larger than those of SHS.

Thinness during adolescence and young adulthood is associated with adverse health consequences in later life, such as osteoporosis and unfavorable pregnancy outcomes, which is regarded as another important health concern different from MS [15,16]. Thus, we also investigated the prevalence of thinness and confirmed the non-negligible proportion in Japanese school girls. Other groups also demonstrated the increasing trend of thinness particularly in young Japanese women aged 15-29 years [17]. Because such a national trend is attributed mainly to an excess desire for slim body shape [18], nutritional education on risks of unhealthy weight loss as well as on benefits of healthy diets would serve to prevent the unfavorable epidemic.

Our group and other groups reveal that urinary excretions of particular minerals and food factors are associated with dietary habit. For instance, increased urinary excretions of Na, K, and taurine significantly correlate with intakes of salty foods, vegetables and fruits, and seafood, respectively [7,8,10,19]. Thus, 24-h urine analysis, in combination with anthropometric and blood chemical analyses, enables us to link between MS risks and dietary habit. Indeed, a significant reduction in serum triglyceride level and increase in serum HDL level were observed in higher urinary K excreters, indicating that the higher group had a lower risk for dyslipidemia. Such an improvement on serum triglyceride level was shown in the higher urinary taurine excretion group. Intakes of vegetables and fruits rich in K, and seafood rich in taurine are likely useful for getting a better lipid profile of Japanese pubertal girls. Furthermore, we confirmed a decreased tendency in insulin level and HOMA-IR in higher urinary taurine excreters, although the differences did not reach statistical significance possibly due to a small studied population. It points out the possibility that taurine could prevent the future development of insulin resistance, a major cause of T2DM and CVDs.

It is widely accepted that the increased intakes of vegetables and fruits or seafood lead to improvement in anthropometric parameters, particularly BP, in adults [20,21]. Hence, one might ask why a significant decrease in anthropometric parameters, such as BP and BMI, was not observed in higher K and taurine excretion groups. Although the reason remains unclear, it could be explained by a small studied population, high rates of non-obese and normotensive participants, or both. Further studies are needed to clarify the correlations between dietary habit and each MS risk in Japanese female adolescents.

## Conclusions

We here demonstrate that hGlc is quite a common MS risk in Japanese JHS and SHS girls aged 13-18 years, whereas overweight and obesity are minor risk factors. These findings indicate that control of plasma glucose level rather than body weight is a crucial task in Japanese pubertal girls. We also confirm that dietary habit rich in K and taurine, namely adequate intakes of vegetables, fruits and seafood, can improve their lipid profile. Given that vegetables, fruits and seafood abundantly contain multifunctional dietary factors other than K and taurine, such as folate and n-3 fatty acids, nutritional education promoting healthy dietary habit would help to improve health status and to prevent the future development of MS in Japanese female adolescents.

## List of abbreviations used

AC: abdominal circumference; BMI: body mass index; BP: blood pressure; CARDIAC study: cardiovascular diseases and alimentary comparison study; CVD: cardiovascular disease; DBP: diastolic blood pressure; dLP: disturbed lipid profile; hGlc: higher plasma glucose; hBP: higher blood pressure; HDL: high-density lipoprotein; HOMA-IR: homeostasis and assessment of insulin resistance; HPLC: high performance liquid chromatography; JHS: junior high school; MS: metabolic syndrome; SHS: senior high school; SBP: systolic blood pressure.

## Competing interests

The authors declare that they have no competing interests.

## Authors' contributions

MM designed research. MI, SA, MT, AH and MM participated in health examination and acquired blood and urine sample. AH performed urinary analysis by HPLC. MI, SA, MT and MM analyzed data. MI and KK wrote the manuscript. All authors read and approved the final manuscript. MM gave final approval of the version to be published.
